# Exploring shared decision-making needs in lung cancer screening among high-risk groups and health care providers in China: a qualitative study

**DOI:** 10.1186/s12885-024-12360-0

**Published:** 2024-05-21

**Authors:** Xiujing Lin, Fangfang Wang, Yonglin Li, Fang Lei, Weisheng Chen, Rachel H. Arbing, Wei-Ti Chen, Feifei Huang

**Affiliations:** 1 School of Nursing, Fujian Medical University, No 1, Xueyu Road, Minhou county, Fujian, Fuzhou, 350108, China; 2 School of Nursing, University of Minnesota, Twin Cities, Minneapolis, MN USA; 3 Department of Thoracic Oncology Surgery, Fujian Medical University Cancer Hospital, Fujian Cancer Hospital, Fuzhou, China; 4 School of Nursing, University of California Los Angeles, Los Angeles, CA 90095 USA

**Keywords:** Early detection of cancer, Lung neoplasms, Qualitative research, Shared decision-making

## Abstract

**Background:**

The intricate balance between the advantages and risks of low-dose computed tomography (LDCT) impedes the utilization of lung cancer screening (LCS). Guiding shared decision-making (SDM) for well-informed choices regarding LCS is pivotal. There has been a notable increase in research related to SDM. However, these studies possess limitations. For example, they may ignore the identification of decision support and needs from the perspective of health care providers and high-risk groups. Additionally, these studies have not adequately addressed the complete SDM process, including pre-decisional needs, the decision-making process, and post-decision experiences. Furthermore, the East-West divide of SDM has been largely ignored. This study aimed to explore the decisional needs and support for shared decision-making for LCS among health care providers and high-risk groups in China.

**Methods:**

Informed by the Ottawa Decision-Support Framework, we conducted qualitative, face-to-face in-depth interviews to explore shared decision-making among 30 lung cancer high-risk individuals and 9 health care providers. Content analysis was used for data analysis.

**Results:**

We identified 4 decisional needs that impair shared decision-making: (1) LCS knowledge deficit; (2) inadequate supportive resources; (3) shared decision-making conceptual bias; and (4) delicate doctor-patient bonds. We identified 3 decision supports: (1) providing information throughout the LCS process; (2) providing shared decision-making decision coaching; and (3) providing decision tools.

**Conclusions:**

This study offers valuable insights into the decisional needs and support required to undergo LCS among high-risk individuals and perspectives from health care providers. Future studies should aim to design interventions that enhance the quality of shared decision-making by offering LCS information, decision tools for LCS, and decision coaching for shared decision-making (e.g., through community nurses). Simultaneously, it is crucial to assess individuals’ needs for effective deliberation to prevent conflicts and regrets after arriving at a decision.

## Background

Low-dose computed tomography (LDCT) is an effective tool for early lung cancer detection and has been proven to enhance survival rates in individuals at high-risk for lung cancer [1, 2]. However, global LDCT usage is limited, with only 2-35% of eligible individuals undergoing screening [3–7], in contrast to 16-68% of eligible candidates undergoing colorectal cancer screening [8]. Improvements in LDCT screening rates for high-risk groups have been modest. The intricate balance between the advantages and risks of LDCT impedes the utilization of lung cancer screening (LCS) [9]. Notably, compared to their non-screened counterparts, high-risk individuals who underwent LDCT had a remarkable 24% decrease in lung cancer mortality [2]. However, the benefits of LDCT come with potential drawbacks, such as radiation-induced cancer, needless examinations, invasive procedures stemming from false positives, overdiagnosis, incidental discoveries, and psychological burdens [10]. These complexities render the LDCT screening decision-making process multifaceted and reliant on personal preferences. Hence, guiding high-risk groups toward well-informed choices regarding LCS is pivotal and represents a substantial mechanism for advancing the secondary prevention of lung cancer.

Shared decision-making is defined as “a collaborative approach for health care providers and patients in making informed health decisions”, which involves considering evidence regarding the benefits and risks of medical options, as well as individuals’ preferences and values [11]. This decision-making process allows both health care providers and individuals as well as their family members to engage in deliberation which leads to identifying the most appropriate decision for the situation [12]. Multiple guidelines strongly recommend shared decision-making as an essential step before patients undergo LDCT. Shared decision-making is also stipulated as a prerequisite for LDCT reimbursement by the Centers for Medicare and Medicaid Services in the United States [13–16]. Regrettably, the utilization of shared decision-making in clinical practice is currently not optimal [17, 18]. Patients do not know what LDCT is, and they often report a lack of about the risks and benefits of LDCT. As a result, patients often have concerns about the risks of LDCT, and health care providers frequently fail to inquire about individuals’ preferences [19]. Consequently, there has been a notable increase in the literature focusing on barriers to shared decision-making from the perspectives of both health care providers and lung cancer high-risk groups. For example, studies have shown that the barriers to shared decision-making include different perceptions about the use of shared decision-making and a lack of time to communicate with providers. However, there are some limitations in terms of methodology and the comparative nature of the studies that focus on LCS shared decision-making. First, previously published studies focused on identifying barriers to shared decision-making and neglected decision support from physicians and patients. For instance, one study found that a lack of professionalism in health care providers is a barrier to shared decision-making, yet no studies have examined specific LCS shared decision-making decision supports for health care providers [19]. Second, current research centers on short-term decision-making experiences, such as cognitive consequences experienced immediately following shared decision-making. However, studies have not adequately addressed the complete shared decision-making process – pre-decisional needs, the decision-making process itself, and post-decision experiences, such as decision regret. Third, the COVID-19 pandemic has introduced a new risk of LDCT usage (exposure to the health-care environment) [20]. The added risk alters the benefit-risk ratio of LDCT under pre-COVID-19 guideline recommendations. Fourth, shared decision-making, developed in Western societies, is rarely discussed in China. The national climate and medical systems of China and Western countries differ greatly [21], and the lack of evidence on LCS shared decision-making in China indicates a need for an assessment of shared decision-making in those who require LDCT.

This study aimed to explore the decisional needs and decision support of shared decision-making for LCS among Chinese high-risk individuals and their health care providers using data collected through in-depth one-on-one interviews.

### Theoretical framework

The Ottawa Decision-Support Framework (ODSF) is an evidence-based conceptual framework that is structured around three key components [22]: (1) assessing decisional needs, such as insufficient knowledge, complex decision types, and limited resources; (2) providing decision support, which encompasses clinical counseling, decision-making tools, and decision coaching; and (3) evaluating decisional outcomes, which includes assessing the quality of the decision-making process and its impact. According to the ODSF, successful decision support should be guided by an assessment of the individual’s knowledge and his/her ability to make his/her own decision to reduce their unmet needs and achieve a final health decision with the support of health care providers and family members. The ODSF has been successfully used within several populations with health needs to guide health decisions and provide decision support [23, 24].

## Methods

### Design

This qualitative study emphasizes the “who, what, and where” of events or experiences [25]. The central research question posed was, “What are the decisional needs and supports of LCS shared decision-making among individuals at high-risk of lung cancer and health care providers?” Consequently, a descriptive qualitative approach was deemed appropriate for exploring the decisional needs and supports for LCS shared decision-making among individuals at high-risk of lung cancer and health care providers [26]. This descriptive qualitative study adhered to the Consolidated Criteria for Reporting Qualitative Studies (COREQ) checklist [27]. Ethical approval for this study was obtained from the ethics committee of Fujian Medical University (Approval No. 2,023,098).

### Inclusion and exclusion criteria

Aligned with the guidelines for the early detection of lung cancer in China [14], the inclusion criteria used for the high-risk group for lung cancer were as follows: (a) aged between 50 and 74 years; (b) had at least one of the following risk factors for lung cancer: a smoking history ≥ 30 pack-years, which includes current smokers or individuals who quit smoking within the last 15 years; prolonged exposure to passive smoking (living or working with smokers for 20 years or more); a history of COPD; a history of occupational exposure to asbestos, radon, beryllium, chromium, cadmium, nickel, silicon, soot, or coal soot for a minimum of 1 year; or a family history of lung cancer; (c) verbal confirmation of undergoing LCS shared decision-making; (d) undergone LDCT within the past 5 years; (e) Able to converse in Mandarin; (f) absence of cognitive or psychological disorders; and (g) willingness to share their personal stories. The exclusion criteria used for the high-risk group for lung cancer were as follows: (a) previous history of lung cancer; and (b) cognitive or psychological disorders (such as depression and anxiety). The inclusion criteria used for health care providers were as follows: (a) certified physicians or nurses; (b) expertise in LCS; and (c) willingness to share their experiences. Healthcare providers who were receiving external training were excluded from participation in the study.

### Qualitative data collection

The data were collected from March 2023 to May 2023. A purposive sampling method was used to identify and recruit individuals at high-risk for lung cancer, as well as local health care providers from five community healthcare centers and two surgical oncology departments of tertiary hospitals. Study flyers provided information on the purpose of the study and the inclusion and exclusion criteria and were distributed to potential participants on site. After participants expressed their interest in the study, they were screened for eligibility to participate and their informed consent was secured. Next, a one-on-one interview was scheduled and a questionnaire was completed by participants to obtain their demographic data (gender, age, residential area, smoking status, etc.). One-on-one interviews were conducted in Mandarin, digitally recorded, with study data stored on a passworded encrypted laptop. Each interview lasted approximately 20 to 40 min. A private room in the clinic was used for all the in-depth interviews.

The interview questions were formulated based on the ODSF and after a comprehensive literature review [28], with extensive discussions among researchers of the study (Feifei Huang, PhD, RN, Professor, specializing in lung cancer prevention and psycho-oncology; Weisheng Chen, MD, specializing in lung cancer prevention, diagnosis and treatment; and Wei-Ti Chen PhD, RN, CNM, FAAN, specializing in intervention design and qualitative data collection). To ensure the acceptability and credibility of the interview guide, the interview questions were pilot tested with four participants in total, including two health care providers and two individuals at high-risk of lung cancer. As a result, some misconceptions regarding the interview questions were identified and subsequently modified. For instance, we replaced the term “decision tools” with “patient decision aids” to help participants to better understand the posed questions. The final interview questions are outlined in Table 1. Tables 2 and 3 summarize key demographic data collected on the high-risk individuals and health care providers, respectively.

**Table 1:** Interview guide

High-risk group	Health care providers
①What is your view on lung cancer screenings	①What is your view on lung cancer screenings
② How did you make a lung cancer screening decision with your doctor?	② How did you help your patients to make a lung cancer screening decision?
③ What difficulties have you encountered in the process of decision-making? How did you solve it?	③ What difficulties have you encountered in decision-making? How did you solve it?
④ How did you feel after making your decision?	④ What kind of feedback have you received after making a decision?
⑤ What factors can help you to make a better screening decision?	⑤ What factors can help high-risk individuals to make better screening decisions?
⑥ What do you think about shared decision-making?	⑥ What do you think about shared decision-making?
⑦ What do you think of patient decision aids?	⑦ What do you think of decision tools?
⑧ Is there anything else you would like to share with me or tell me?	⑧ Is there anything else you would like to share with me or tell me?

**Table 2:** Demographic characteristics of the high-risk participants (*n* = 30)

No.	Gender	Age (years)	Place of residence	Education level	Monthly income (USD)	Medical insurance type
1	Male	50	Rural	Middle school	>715	BMIS-UWR
2	Female	55	Rural	Middle school	>715	BMIS-UWR
3	Male	55	Urban	High school	>715	BMIS-UWR
4	Female	53	Urban	Bachelor	>715	BMIS-UWR
5	Female	50	Rural	Bachelor	>715	BMIS-UWR
6	Male	58	Rural	Bachelor	<500	BMIS-UWR
7	Female	55	Rural	Bachelor	>715	BMIS-UWR
8	Female	54	Urban	High school	>715	BMIS-UWR
9	Male	57	Urban	Bachelor	>715	BMIS-UWR
10	Female	51	Rural	High school	<500	BMIS-UWR
11	Female	54	Rural	Bachelor	500–715	BMIS-UWR
12	Male	52	Rural	Middle school	<500	BMIS-UWR
13	Female	53	Rural	Bachelor	<500	BMIS-UWR
14	Female	55	Urban	High school	500–715	BMIS-UWR
15	Male	65	Urban	High school	500–715	BMIS-UWR
16	Female	67	Rural	High school	500–715	BMIS-UWR
17	Female	69	Rural	Bachelor	<500	BMIS-UWR
18	Male	67	Urban	High school	<500	BMIS-UWR
19	Male	73	Urban	Middle school	500–715	BMIS-UWR
20	Female	71	Urban	High school	<500	BMIS-UWR
21	Male	71	Urban	High school	<500	BMIS-UWR
22	Female	74	Urban	Middle school	>715	BMIS-UWR
23	Female	72	Rural	High school	>715	BMIS-UWR
24	Female	60	Rural	High school	>715	BMIS-UWR
25	Female	68	Rural	High school	<500	BMIS-UWR
26	Male	64	Urban	Bachelor	>715	BMIS-UWR
27	Female	72	Urban	Middle school	<500	BMIS-UWR
28	Female	63	Urban	High school	500–715	BMIS-UWR
29	Female	64	Urban	High school	500–715	BMIS-UWR
30	Male	66	Rural	High school	500–715	BMIS-UWR

*BMIS-UWR* Basic medical insurance system for urban workers and residents

**Table 3:** Demographic characteristics of health care provider participants (*n* = 9)

No.	Gender	Age (year)	Place of residence	Education level	Occupation	Work experience (year)	Career level	Workplace
1	Female	34	Urban	Bachelor	Nurse	12	Mid	Community hospital
2	Female	41	Urban	Bachelor	Nurse	18	Senior	Grade A tertiary hospital
3	Female	46	Urban	Bachelor	Nurse	26	Mid	Grade A tertiary hospital
4	Female	45	Urban	Bachelor	Nurse	27	Mid	Grade A tertiary hospital
5	Male	25	Urban	Postgraduate	Physician	2	Junior	Grade A tertiary hospital
6	Male	27	Urban	Postgraduate	Physician	2	Junior	Grade A tertiary hospital
7	Female	35	Urban	Bachelor	Physician	15	Mid	Community hospital
8	Female	36	Urban	Bachelor	Physician	16	Mid	Community hospital
9	Male	42	Rural	Middle school	Physician	18	Mid	Community hospital

The sample size was determined by data saturation, that is, recruitment ended at the point where no new themes emerged from the participants’ experiences [29]. Data saturation was reached at approximately the twenty-seventh in-depth interview with a high-risk lung cancer individual, with another three high-risk lung cancer individuals being interviewed to ensure that the data reached complete saturation. Data saturation was reached at approximately the seventh in-depth interview with healthcare providers, with another two healthcare providers interviewed to ensure data saturation.

### Data analysis

**Fig. 1:**
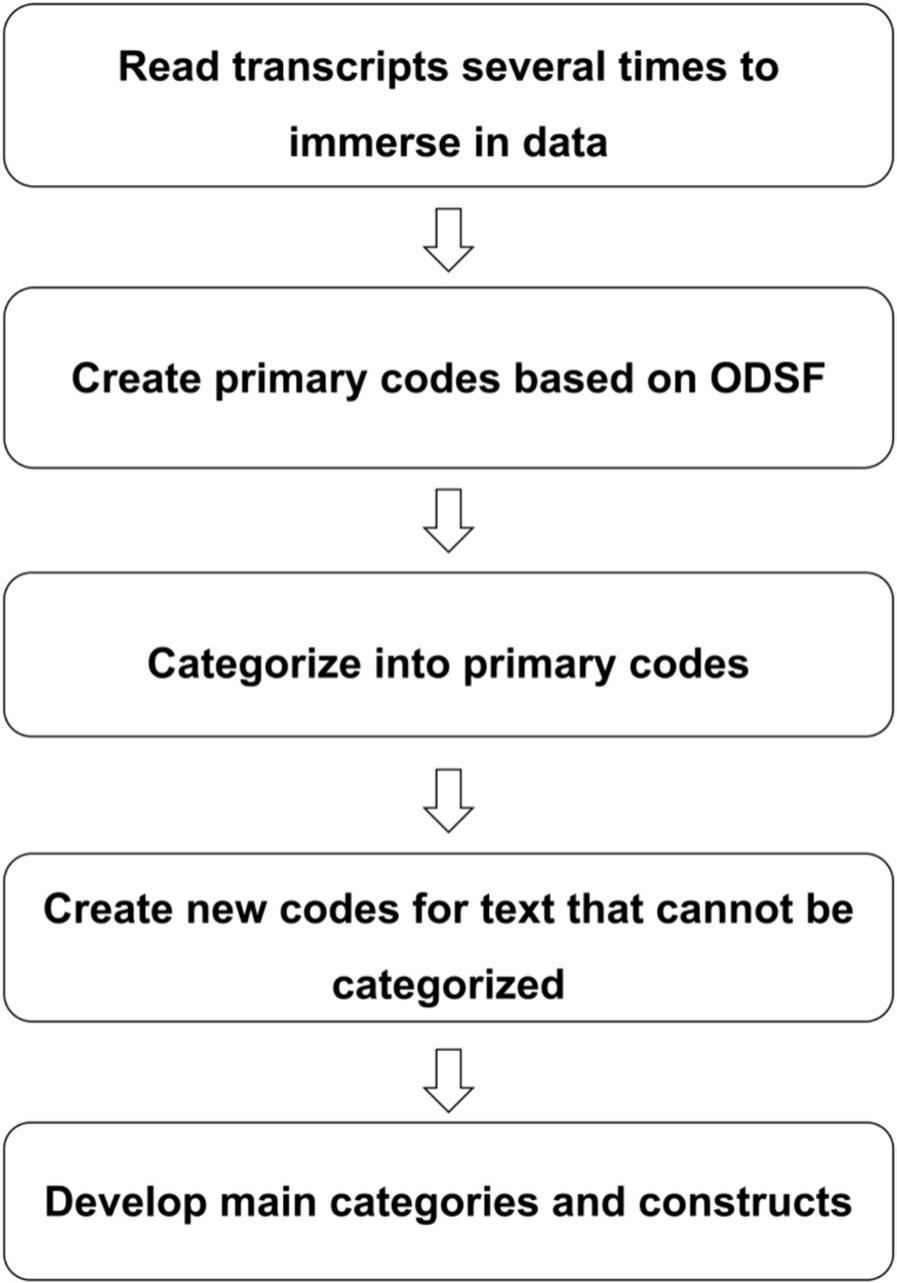
Directed content analysis flowchart

Since the interviews were conducted in Mandarin, a bilingual coding technique was used to keep the data in the original Chinese format, and the coding assignments were in English (e.g., decision negotiation). To ensure accuracy and minimize potential translation errors, two bilingual researchers (Chinese and English) reviewed and confirmed the translations [30]. The process of data analysis began with data collection. To analyze the data, content analysis was guided by the ODSF and Nvivo software version 12 was used [31]. The classification of themes was performed both inductively (derived from the quotes of research participants) and deductively (derived from the ODSF theoretical framework) under the principle of complementarity. The detailed steps of the data analysis process are illustrated in Fig. 1.

### Trustworthiness

Credibility, dependability, confirmability and transferability were employed to assure the trustworthiness of this study’s findings [32]. To enhance credibility, the researcher dedicated ample time to establishing meaningful interactions with the participants, thereby building trust for effective data collection. Regarding dependability, two researchers cross-checked and rectified codes that did not precisely reflect participants’ perspectives. Furthermore, an audit trail and reflexivity techniques were used during the data analysis process, which included tracking the interview and data analysis notes and memos. To ensure confirmability, the supervisor reviewed and selected quotations, codes, and categories, thereby validating the accuracy of the coding process. In terms of transferability, participants were purposefully selected from both urban and rural areas to incorporate a wide range of perspectives. Herein, a comprehensive description of the entire research process is presented to facilitate reproducibility of the study.

## Results

Out of a total of 44 participants consented, five participants (4 high-risk individuals and 1 health care provider) dropped out of the study due to their busy schedules and lack of interest in participating. A total of 39 eligible volunteers composed the study sample. Among them, 30 individuals were classified as at high-risk for lung cancer with an average age of 61.27 ± 7.92 years, while nine health care providers had an average age of 36.78 ± 7.45 years. Five health care provider participants specialized in lung cancer prevention, diagnosis, and treatment, and four specialized in general medical education and community cancer screening education. Detailed demographic information on the participants can be found in Tables 2 and 3.

**Fig. 2:**
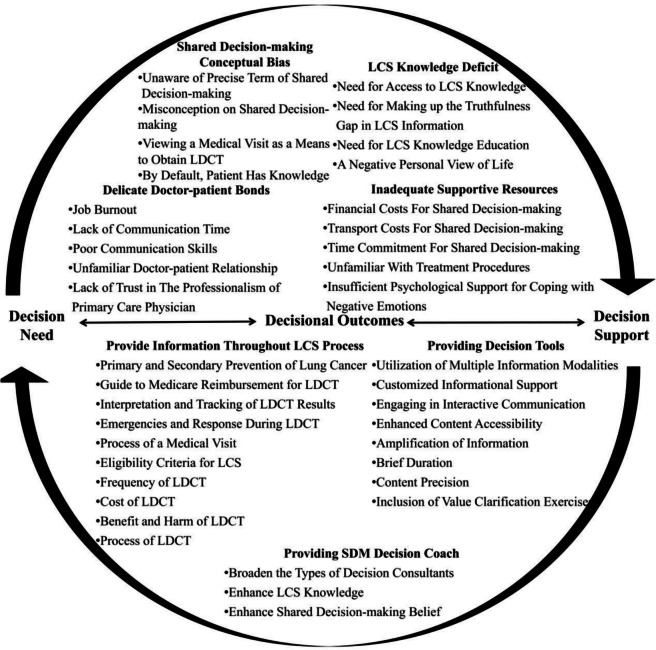
Participants’ viewpoints on shared decision-making based on ODSF

A total of 546 unique codes related to LCS shared decision-making were identified. Following the framework of the ODSF, participants’ decisional needs and supports for shared decision-making were categorized (refer to Fig. 2; Table 4).

**Table 4:** Component themes of the Ottawa decision support framework

Component	Theme	Subcategory	Illustrative Quote
Decision need	LCS knowledge deficit	Need for Access to LCS Knowledge	“The ideal approach to acquire knowledge would be to consult my doctor for verification, but due to time constraints, they can’t address all queries. As a result, I lack the access to LCS knowledge.” H13
		Need for Making up the Truthfulness Gap in LCS Information	“It seems that I do not find an effective way to tell the truth of the medical information. At times, I cross-check the credibility of information across various online platforms. When there’s inconsistency, I become skeptical about its authenticity.” H13
		Need for LCS Knowledge Education	“Every now and then, my own knowledge might not be enough to navigate through medical decisions, mainly because there are significant professional barriers in the field.” H9
		A Negative Personal View of Life	“The choice to move forward should consider both their family’s financial situation and their outlook on life. Some people believe in destiny, while others put their trust in science.” H3
	Inadequate supportive resources	Financial Costs for SDM	“Before I started SDM with my doctor, I need to pay for the fee of outpatient care. Sometimes, it takes several visits to make a final decision, the outpatient care fee cannot be ignored.” H13
		Transport Costs for SDM	“The top-tier Grade A tertiary hospitals are mainly located in the city center. It takes a long time to drive for SDM. ” H8
		Time Commitment for SDM	“Hospital visits are inconvenient. Taking a day off work and enduring lengthy queues for medical consultations and tests feels like a time-consuming ordeal.” H12
		Unfamiliar with Treatment Procedures	“The hospital was unfamiliar territory for me, and I had a hard time understanding the treatment procedures. Finding a doctor in the complex process proved to be quite challenging.” H8
		Insufficient Psychological Support for Coping with Negative Emotions	“Since I smoke, I’m always scared of getting bad test results. If the results are bad, it’s just really scary, I don’t think I have the sanity to make shared decisions with my doctors. I need help.” H11 “I struggle with claustrophobia, and every time I have a test, I feel really trapped. It would be difficult for me to have shared decision making when I have a claustrophobia. It felt like my mind was blank.” H12
	SDM conceptual bias	Unaware of Precise Term of SDM	“I believe that when it comes to professional matters, it’s best to rely on trained professionals. Most patients don’t have expert medical knowledge, and even if they do, they might be hesitant about certain exams. That, in my opinion, doesn’t do much good for their health.” M8
		Misconception on Shared Decision Making	“I think shared decision-making means thoroughly informing those in high-risk groups about the pros and cons of a particular exam and ultimately letting them make the call.” M5 “We’re already implementing shared decision-making. For example, when patients aren’t familiar with their treatment plans, helping them fill out an informed consent form gives us a chance to have a conversation with them and make sure they really understand their treatment plan. To me, that’s a clear example of shared decision-making.” M3
		Viewing a Medical Visit as a Means to Obtain LDCT	“When we suggest undergoing a medical examination, doctors might assume that this visit is a necessary step for patients to get a chance to be examined, not a step for shared decision-making. As a result, they may believe that there’s no necessity for patient education.” H13
		By Default, Patient Has Knowledge	“When a doctor suggests a medical examination, they might assume that I’ve thoroughly grasped all the relevant information before making a decision. So, they might think patient education isn’t necessary. ” H13
	Delicate Doctor-patient Bonds	Job Burnout	“I believe that doctor burnout contributes to their reluctance to discuss lung cancer screening with patients.” H9
		Lack of Communication Time	“I have a very demanding work schedule, which leaves me with limited time to engage in shared decision-making with doctors.” H12 “Due to the demanding nature of my work, and sometimes the high number of patients, we often find ourselves lacking the time needed for shared decision-making.” M9
		Poor Communication Skills	“Effective communication is essential for both doctors and patients. The doctor’s ability to convey information and the patient’s capacity to express their needs are crucial. Insufficient communication skills represent a challenge for both parties.” M6
		Unfamiliar Doctor-patient Relationship	“Building trust is not a simple task. When patients and I have a strong connection and they trust us enough to share their true thoughts, it significantly reduces barriers to shared decision-making. On the other hand, some doctors who aren’t deeply connected with the community may struggle to gain patients’ trust, leading to communication challenges that hinder shared decision-making.” M2
		Lack of Trust in the Professionalism of Primary Care Physician	“Personally, I believe that the expertise of doctors in county-level hospitals may not be as advanced, which affects my level of trust in them. I tend to find doctors in top-tier tertiary hospitals to be more credible.” H12
Decision support	Provide Information Throughout LCS Process	Primary and Secondary Prevention of Lung Cancer	“I think it’s really important to focus on both preventing lung cancer in the first place and catching it early. This way, people can understand better what’s at risk, especially if they smoke.” M5
		Eligibility Criteria for LCS	“Information about eligibility for lung cancer screening should be provided, along with guidance on how to interpret the results and what steps to take next.” H7
		Frequency of LDCT	“I’m not sure how often it’s recommended to get a lung cancer screening.” H7
		Benefit and Harm of LDCT	“I want to learn about the cost, benefits, and potential risks of lung cancer screening. While hospitals often emphasize the benefits, they rarely discuss the possible harms. This could lead some individuals to regret their decision after undergoing the test.” H12
		Cost of LDCT	“I want to learn about the cost, benefits, and potential risks of lung cancer screening. While hospitals often emphasize the benefits, they rarely discuss the possible harms. This could lead some individuals to regret their decision after undergoing the test.” H12
		Process of LDCT	“It is disconcerting to undergo LDCT without an advanced discussion. Doctors should explain how lung cancer screening is conducted, as well as provide precautions to take before and after the procedure.” H3
		Medical Visit Provider Steps	“In addition to presenting information about lung cancer screening, practical knowledge is also necessary. Teaching us how to schedule appointments, undergo examinations, and navigate the reimbursement process would be valuable.” H5
		Emergencies and Response during LDCT	“I’m hoping the doctor can take the time to explain important details, like possible scenarios that might come up during the lung cancer screening.” H9
		Interpretation and Tracking of LDCT Results	“We should give folks clear info on who’s eligible for lung cancer screening, and also guide them on how to understand the results and what to do next.” H10 “What I’m most curious about is what happens after we get the results. Specifically, whether they’re good or bad, when I should come back for a recheck, and what I should be mindful of in my everyday life.” H9
		Guide to Medicare Reimbursement for LDCT	“In addition to presenting information about lung cancer screening, practical knowledge is also necessary. Teaching us how to schedule appointments, undergo examinations, and navigate the reimbursement process would be valuable.” H12
	Providing SDM Decision Coach	Enhance SDM Belief	“In China, shared decision-making isn’t commonly practiced. Many physicians here may not be familiar with the concept, even though it’s something they should consider adopting. Personally, I strongly believe in the importance of implementing shared decision-making.” H6 “However, there are several factors that can make it challenging for patients to fully engage in shared decision-making process. For example, they are more dependent on doctors in the decision-making process due to their knowledge limitation. They never thought they could be decision-makers.” M5
		Enhance LCS Knowledge	“Before participating in shared decision-making, I’d like to gain some basic medical knowledge.” H4 “The quality of shared decision-making is closely tied to the professionalism of physicians. Some doctors might not have a very high level of professionalism, which can impact their ability to provide expert opinions. So, for shared decision-making to be effective, it’s essential for physicians to have a strong professional background.” H13
		Broaden the Types of Decision Consultants	“It’s important to involve community health workers in shared decision-making for a couple of reasons. Firstly, we tend to establish a strong rapport with patients, and they often trust us more compared to clinicians. Additionally, we have the advantage of spending more time communicating with patients, which makes us better suited to facilitate shared decision-making.” M9
	Providing Decision Tools	Utilization of Multiple Information Modalities	“Combining pictures, text, and videos is essential. Given that many high-risk groups are elderly, using videos as a medium can enhance their understanding and accommodate their learning within fragmented time periods. For example, videos can be conveniently viewed during public transportation. Decision tools primarily composed of text tend to be overly complex for patients.” M5
		Customized Informational Support	“Decision tools need to have a tailored function. They should provide me with my specific lung cancer risk and offer the necessary lung cancer prevention information that pertains to me.” H10
		Engaging in Interactive Communication	“Interaction holds great significance. The presence of doctors for guidance is crucial when using a decision tool.” H9
		Enhanced Content Accessibility	“I believe that highly advanced medical knowledge should be presented to the public in a welcoming and easily understandable manner.” H4
		Amplification of Information	“When I have a genuine interest in medical knowledge, I find the superficial information provided by decision tools unsatisfying. Instead, I have a strong desire to pursue ongoing learning.” H4
		Brief Duration	“If the process of using decision tools takes up a lot of time, a considerable number of users may abandon the process or not engage with it seriously.” M7
		Content Precision	“Involving medical professionals in the development of decision tools is highly advisable, as it lends authority to the tool.” H13
		Inclusion of Value Clarification Exercises	“Value clarification exercises hold significant value, as they can mitigate the struggle experienced by decision makers. By engaging in a brief exercise aimed at clarifying individual screening preferences, these exercises can effectively reduce the challenges individuals face.” H12\

*H* High-risk group of lung cancer patients, *M* Medical health care provider, *LCS* Lung cancer screening, *LDCT* Low-dose computed tomography, *SDM* Shared decision making

### Decisional needs

We identified four categories related to the theme of decisional needs, including LCS knowledge deficits, inadequate supportive resources, shared decision-making conceptual bias, and delicate doctor-patient bonds.

#### Theme 1: LCS knowledge deficit

Many high-risk study participants expressed that they did not have access to reliable and authoritative medical information. Many of the high-risk participants shared their inability to access LCS-related information and their limited capacity to distinguish accurate LCS information from misinformation. Furthermore, participants mentioned that a negative personal view of life influenced their active engagement in shared decision-making with health care providers and/or family, which diminished their comprehensive understanding of LCS.

“Some people are negative, they believe God’s will can decide everything, so when they faced a decision, they will ask the gods instead of making a decision according to their actual situation” H13 (high-risk individual, female, 53 years-old).

#### Theme 2: inadequate supportive resources

Participants emphasized that shared decision-making was hindered by financial, transportation and time-related barriers to hospital visits. Furthermore, unfamiliarity with the process of seeking medical treatment also presented an obstacle to shared decision-making. Notably, participants expressed negative emotions related to the LDCT test which influenced their shared decision-making. In particular, the LDCT process was not well received by individuals who had claustrophobia. Participants described feeling claustrophobic during the process of the imagological examination. The requirement for patients to lie flat during the examination, combined with the confined and dim space, can lead to feelings of depression and suffocation. Additionally, the machine’s noise and concerns about potential risks (such as radiation and false positives) from having LDCT scans may have heightened patients’ negative emotions and fears.

“Since I smoke, I’m always scared of getting bad test results. If the results are bad, it’s just really scary, I don’t think I have the sanity to make shared decisions with my doctors. I need help.” H11 (a high-risk individual, female, 54 years-old).

“I struggle with claustrophobia, and every time I have a test, I feel really trapped. It would be difficult for me to have shared decision-making when I have a claustrophobia. It felt like my mind was blank.” H12 (a high-risk individual, male, 52 years-old).

Several participants mentioned experiencing anxiety regarding the test results. They expressed their apprehension about potential adverse outcomes and indicated that this anxiety affected their ability to engage in shared decision-making with their doctors. Moreover, after experiencing claustrophobia, some participants expressed that they felt an inability to make shared decisions with their doctors in a rational manner.

#### Theme 3: Shared decision-making conceptual bias

Some participants mentioned that they were not familiar with the specific term ‘shared decision-making’. Health care providers shared the perspective that excessive communication with the high-risk group about their condition might lead to a refusal of subsequent treatment, potentially jeopardizing their health.

“I believe that when it comes to professional matters, it’s best to rely on trained professionals. Most patients don’t have expert medical knowledge, and even if they do, they might be hesitant about certain exams. That, in my opinion, doesn’t do much good for their health.” M8 (a general practitioner, female, 36 years-old).

Additionally, participants had misconceptions about shared decision-making. For example, health care providers had misconceptions about shared decision-making in LDCT screenings – some believed that shared decision-making meant merely providing information about the benefits and risks of LDCT; others confused the concepts of informed consent and shared decision-making all together; and a few providers viewed encouraging high-risk groups to conduct LDCT screening to be a part of shared decision-making. Some participants believed shared decision-making to be merely a procedural step to schedule a test appointment.

“I think shared decision-making means thoroughly informing those in high-risk groups about the pros and cons of a particular exam and ultimately letting them make the call.” M5 (a physician specialist, male, 25 years-old).

“When we suggest undergoing a medical examination, doctors might assume that this visit is a necessary step for patients to get a chance to be examined, not a step for shared decision-making. As a result, they may believe that there’s no necessity for patient education.” H13 (a high-risk individual, female, 53 years-old).

#### Theme 4: delicate doctor-patient bonds

Both health care providers and high-risk individuals emphasized that time constraints pose a significant barrier to shared decision-making. Some participants noted that doctors, who often express concerns about work-related burnout, were hesitant to provide comprehensive information about LDCT.

“I believe that doctor burnout contributes to their reluctance to discuss lung cancer screening with patients.” H9 (a high-risk individual, male, 57 years-old).

Furthermore, health care providers and participants encountered challenges with communication. Health care providers struggled to simplify complex information for easy understanding, while participants had difficulty clearly expressing their needs.

“Effective communication is essential for both doctors and patients. The doctor’s ability to convey information and the patient’s capacity to express their needs are crucial. Insufficient communication skills represent a challenge for both parties.” M6 (a physician specialist, male, 27 years-old).

Participants also mentioned that they were hesitant to express their thoughts to doctors whom they do not know well.

“Building trust is not a simple task. When patients and I have a strong connection and they trust us enough to share their true thoughts, it significantly reduces barriers to shared decision-making. On the other hand, some doctors who aren’t deeply connected with the community may struggle to gain patients’ trust, leading to communication challenges that hinder shared decision-making.” M2 (a nurse in grade A tertiary hospital, female, 41 years-old).

Others believe that the professional competence of doctors plays a pivotal role in shared decision-making in LCS. People often opt for doctors from tertiary hospitals who were perceived to have a higher level of professionalism, which is conducive to shared decision-making.

“Personally, I believe that the expertise of doctors in county-level hospitals may not be as advanced, which affects my level of trust in them. I tend to find doctors in top-tier tertiary hospitals to be more credible.” H12 (a high-risk individual, male, 52 years-old).

### Decision support

Three categories related to the theme of decision support were identified: provide information throughout the LCS process, providing a shared decision-making coach, and provide decision tools.

#### Theme 1: provide information throughout the LCS process

Participants shared that they would like to know information about LDCT before and after undergoing the screening test. Desired information prior to screening included: eligibility criteria for LCS; benefits and risks of LDCT, the LDCT process itself, primary and secondary prevention of lung cancer, the cost of LDCT, potential emergencies and appropriate responses during LDCT, guidelines for Medicare reimbursement related to LDCT, and the medical visit steps. Most participants wanted information after the screening to include the interpretation and monitoring of LDCT results as well as the recommended frequency of LDCT.

#### Theme 2: providing a shared decision-making decision coach

Several participants said that it is necessary to enhance shared decision-making beliefs to better support the decision-making process for LCS, which is inherently a preference-sensitive decision.

“In China, shared decision-making isn’t commonly practiced. Many physicians here may not be familiar with the concept, even though it’s something they should consider adopting. Personally, I strongly believe in the importance of implementing shared decision-making.” H6 (a high-risk individual, male, 58 years-old).

High-risk individuals emphasize the importance of establishing a foundation for knowledge before engaging in shared decision-making. Participants advocated for a basic understanding of medical concepts, with decision counselors possessing specialized medical expertise.

“Before participating in shared decision-making, I’d like to gain some basic medical knowledge.” H4 (a high-risk individual, female, 53 years-old).

Due to time and energy constraints, clinicians found it challenging to engage in shared decision-making. However, the community doctors in our study stated that they had more time to communicate and share opinions and that their closer patient-provider relationships could facilitate the shared decision-making process in China.

“We only present the benefit and harm of LDCT briefly. We don’t have enough time to describe these in more detail. You know, lung cancer pathology and knowledge of imaging are too complex for high-risk individuals of lung cancer. For individuals who don’t have professional backgrounds, it is impossible for them to understand totally, what we can do is try to get them to understand as much as possible in a limited time.” M5 (a doctor in grade A tertiary hospital, male, 25 years-old).

“It’s important to involve community health providers in shared decision-making for a couple of reasons. Firstly, we tend to establish a strong rapport with patients, and they often trust us more compared to clinicians. Additionally, we have the advantage of spending more time communicating with patients, which makes us better suited to facilitate shared decision-making.” M9 (a general practitioner, male, 42 years-old).

#### Theme 3: providing decision tools

Participants expressed the need for decision tools and made several suggestions for decision tools to better cater to diverse groups. Decision tools are instruments that aid users in clarifying the congruence between their decisions and their individual values by presenting relevant options along with their associated benefits and potential drawbacks. Through the use of decision tools, users are assisted in arriving at clear, high-quality decisions.

The participants had several suggestions for providing decision tools. First, various information modalities such as videos, images, and written content should be integrated into tools to accommodate varying education levels and preferences. Second, tailored information that aligns with LCS decision-making is preferred. Third, a three-way interaction model involving patients, decision tools, and health care providers could enhance effectiveness. Fourth, medical knowledge should be presented in a comprehensible manner to improve accessibility. Additionally, access to more detailed information is necessary. Fifth, the time spent using decision tools should be less than 20 min to prevent impatience. Sixth, most participants emphasized addressing credibility concerns, through incorporating medical professionals into the tool’s development team, emphasizing authoritative sources, and involving experts from reputable hospitals. Finally, most participants acknowledged that value clarification exercises should be integrated to help users articulate their personal screening preferences to ensure a comprehensive approach to decision support.

## Discussion

Shared decision-making plays a crucial role in enhancing the understanding of LCS and LDCT in high-risk groups. Shared decision-making can also establish realistic expectations for health outcomes and ultimately improve decision-making for the best treatment or screening option [33]. This qualitative study provides insights into the decisional needs and necessary support for shared decision-making in LDCT screening, from the perspectives of health care providers and high-risk individuals in China. Specifically, LDCT screening decisions should evaluate the knowledge, availability of supportive resources, health care providers’ understanding of shared decision-making concepts, and quality of doctor-patient relationships. At present, both providers and screeners require decision support surrounding LDCT information and need shared decision-making coaching to effectively arrive at a decision. This study finding is valuable for shaping the design of future interventions that aim to facilitate decision-making and has the potential to increase the use of LDCT screening in Chinese society.

Our findings also contribute to the classification refinement of the ODSF. Regarding LCS knowledge, we have observed that high-risk groups not only lack specific knowledge of LCS, but also face challenges accessing relevant information and struggle with their capacity to distinguish accurate LCS information from misinformation. Previous multimodel public health interventions have focused on education related to specific LCS knowledge and ignored the need to access correct information, insufficiently addressing the needs of populations at high-risk of lung cancer [34]. Therefore, in addition to limited knowledge, limited access to information and lack of identification undermine the contributions of high-risk groups in shared decision-making.

In terms of support and resources, it is essential to consider not only conventional limitations such as financial and health system resources, but also the psychological well-being of high-risk populations. The proportion of smokers is greater among those at high-risk for lung cancer than among those at high-risk for other types of cancers (such as breast cancer and colorectal cancer) [35]. Being a smoker can affect the execution of shared decision-making due to perceived stigma, lung cancer fatalism, and heightened levels of worry and fear of contracting lung cancer [35]. Additionally, concerns about potential risks associated with LDCT serve as a barrier to the shared decision-making process with health care providers [9].

Our findings provide new insights into the core constructs of decisional needs, including awareness of shared decision-making and doctor-patient bonds. Additionally, shared decision-making awareness studies have demonstrated that bias can lead to differences in individual preferences, which can hinder the initiation of shared decision-making and result in higher levels of decision conflict [36]. Additionally, studies have shown that poor doctor-patient communication can lead to low-quality shared decision-making. For example, dismissive clinicians who dominate decision-making encounters, use negative verbal or nonverbal cues, or fail to respect patients’ concerns have been shown to act as barriers to shared decision-making for many patients [37]. Conversely, clinicians who strive to understand individual needs and preferences can foster a sense of partnership and facilitate their involvement in shared decision-making processes [38]. It has also been found that allocating limited time for consultations as well as poor communication skills results in ineffective shared decision-making [39]. Limitations in skill and time can impede the ability to be fully informed by health care providers, to process and reflect on the information received, and to engage in meaningful discussions between providers and individuals [37]. Furthermore, the presence of trust is identified as a facilitator of shared decision-making. Establishing a trusting relationship with health care providers encourages patients to feel more comfortable asking questions, sharing personal information, and discussing their concerns [39].

Currently, the use of shared decision-making in clinical practice is suboptimal in China [11]. Fortunately, our study provides potential mitigation strategies. First, the need for comprehensive decision tools that appeal to diverse groups of patients was emphasized by both high-risk groups and health providers. A decision tool can furnish information, facilitate patient-doctor dialog, and enhance therapeutic outcomes [33]. However, the availability of decision tools for LCS is limited and their applications are less than ideal, partly due to their failure to be tailored to personal needs. For instance, most LCS decision tools are presented as single-page materials or premade videos, which may not fully address participants’ needs. Our findings highlight the demand for personalized decision tools for LCS in China. Second, some participants suggested that decision counselors should not be limited solely to clinicians; community health care providers can also serve as counselors for decision-making. This aligns with the concept that shared decision-making requires multisectoral collaboration [40]. Community nurses in particular, share similar ethnic, linguistic, and geographic backgrounds with the residents they serve compared to other nurses. Consequently, they are more likely to encounter high-risk populations in the community [41]. Additionally, due to the nature of their work, they have more time to engage in shared decision-making discussions with high-risk groups. Research has revealed that community nurses, in their roles as coordinators, educators, researchers, navigators, and practitioners, can play multidimensional roles essential for leading successful LCS [42]. Hence, future research should actively promote the development of community nurses as counsellors for LCS to alleviate the burden on hospital-based physicians. Third, both health care providers and high-risk groups should receive education on shared decision-making. Our findings reveal that both sides still possess a vague understanding of shared decision-making, often conflating it with informed consent (patient-led) and paternalism (physician-led) models. Unlike in Western countries, humanistic medicine education in China is lacking, resulting in an inadequate grasp of patient-centered medical-ethical principles among health providers and patients [21]. Future interventions in China should emphasize humanistic medicine to establish the foundation of shared decision-making.

Our findings are rooted in Chinese culture, which, along with broader Asian cultural influences, places a significant emphasis on Confucianism and sociocultural values such as family support, care, and respect for familial hierarchy and authority [43]. Therefore, the insights provided by this paper may be applicable to other Asian countries. Despite the rapid development of SDM research in the West, the actual implementation of SDM in clinical practice is not as favorable [44]. One contributing factor is that highly developed patient decision aids often overly focus on standardized processes, deviating from a more humanistic approach that can be applied universally [44]. Moreover, the ongoing wave of globalization has resulted in increasingly multicultural societies, necessitating a broader scope of SDM coverage that includes individuals from diverse cultural backgrounds. Therefore, avoiding cultural stereotypes and actively inquiring about patients’ preferences become especially crucial. The results of our study contribute valuable insights into individual decisional needs and decision support from the perspectives of both individuals at high-risk for lung cancer and health care providers. These perspectives can assist patient decision aids in avoiding excessive standardization. Simultaneously, the perspective embedded in our findings is well-suited to accommodate the multicultural nature of Western countries. Future studies should seek to bridge the gap in SDM between Eastern and Western contexts.

## Limitations

There are several limitations in this study. First, since the high-risk lung cancer individuals in our study did not undergo LCS shared decision-making recently, their views on LCS shared decision-making may have been subject to recall bias. Second, all study participants were from Fujian Province, which is a southeastern province in China. It is possible that recruitment from a broader geographical area may have led to a wider range of perspectives and experiences and thus influenced the point at which data saturation was reached. Third, as a qualitative, in-depth interview study, generalizations of findings to a larger population are not possible. Future quantitative studies should explore decision-making experiences among a broad range of high-risk groups and health care providers in China to enhance data triangulation and thus, the credibility and reliability of the study’s findings.

## Conclusions

Guiding high-risk groups toward well-informed choices regarding LCS represents a substantial gain toward advancing secondary prevention of lung cancer. This descriptive qualitative study offers valuable insights into decision-making regarding LDCT screening among Chinese high-risk groups and their health care providers. The findings from this study highlight the decisional needs and decision support for shared decision-making for LCS using the ODSF conceptual framework. Future studies should target intervention development to offer decision support by evaluating individuals’ decisional needs, enabling them to make choices confidently, and with minimal conflict and decisional regret. In addition, this study may also serve as a starting point for the development of more effective decision tools for LDCT screening.

## Data Availability

The de-identified datasets used and/or analysed during the current study available from the corresponding author on reasonable request.

## Abbreviations

LDCT: Low-dose computed tomography
LCS: Lung cancer screening
ODSF: The Ottawa Decision-Support Framework
